# Understanding the Pathophysiology of Portosystemic Shunt by Simulation Using an Electric Circuit

**DOI:** 10.1155/2016/2097363

**Published:** 2016-10-27

**Authors:** Moonhwan Kim, Keon-Young Lee

**Affiliations:** Department of Surgery, Inha University School of Medicine, Incheon 400-711, Republic of Korea

## Abstract

Portosystemic shunt (PSS) without a definable cause is a rare condition, and most of the studies on this topic are small series or based on case reports. Moreover, no firm agreement has been reached on the definition and classification of various forms of PSS, which makes it difficult to compare and analyze the management. The blood flow can be seen very similar to an electric current, governed by Ohm's law. The simulation of PSS using an electric circuit, combined with the interpretation of reported management results, can provide intuitive insights into the underlying mechanism of PSS development. In this article, we have built a model of PSS using electric circuit symbols and explained clinical manifestations as well as the possible mechanisms underlying a PSS formation.

## 1. Introduction

Portosystemic shunt (PSS) is a common condition and usually follows portal hypertension or liver trauma, including iatrogenic injury [1–3]. However, congenital or spontaneous PSS can also occur and presents diagnostic along with management challenges [3]. The definition and classification of PSS are in a chaotic status in respect to its cause, location, and anatomical inflow/outflow vessels. This situation probably arose because of lacking consensus, due to most of the relevant literature being composed of case reports or small series [4, 5]. The blood flow is basically similar to an electric current, in that it is determined by pressure difference and resistance, governed by Ohm's law [6]. In this article, we tried to develop a model of PSS using electric circuit symbols and applied it to the interpretation of the reported management results of PSS. Also, we suggested that PSS can be classified according to two distinct underlying mechanisms.

## 2. Materials and Methods

### 2.1. Developing an Electric Circuit Model of PSS

**Figure 1:**
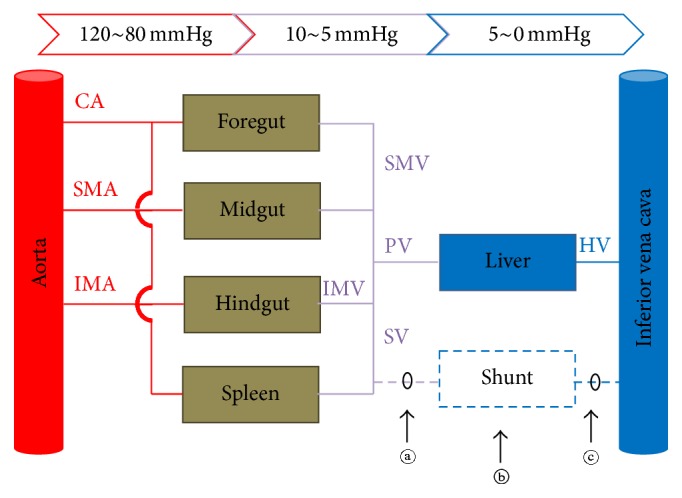
Schematic diagram of abdominal vascular connections ignoring spatial relations. The portal system is depicted by purple lines. Any abnormal connection between the portal system and the systemic veins can form a shunt circuit (dotted line). Note that collaterals between aortic branches are omitted. CA: celiac artery. SMA: superior mesenteric artery. IMA: inferior mesenteric artery. IMV: inferior mesenteric vein. SMV: superior mesenteric vein. PV: portal vein. SV: splenic vein. HV: hepatic vein. *ⓐ*, *ⓑ*, and *ⓒ*: possible shunt occlusion sites.

**Figure 2:**
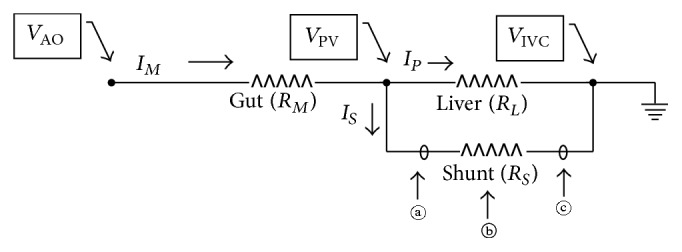
Electric circuit diagram simulating a portosystemic shunt. *V*_AO_: aortic pressure. *V*_PV_: portal pressure. *V*_IVC_: systemic venous pressure. *I*_*M*_: mesenteric flow. *I*_*P*_: portal flow. *I*_*S*_: shunt flow. *R*_*M*_: resistance of mesenteric vessels. *R*_*L*_: resistance of intrahepatic portal vasculature. *R*_*S*_: resistance of shunt. *ⓐ*, *ⓑ*, and *ⓒ*: possible shunt occlusion sites.

The schematic diagram of the splanchnic circulation is presented in Figure 1. By representing the blood flow as an electric current and the vascular resistance of intraabdominal organs as resistors, the intraabdominal vascular system can be further simplified using electric symbols (Figure 2). We assumed that the aortic pressure (*V*_AO_) and mesenteric vascular resistance (*R*_*M*_) are constant and the systemic venous pressure (*V*_IVC_) is approaching zero (grounded).

### 2.2. Literature Review

We have reviewed the English literature articles that were published between 1999 and 2014 and searched case reports or series which presented the management results of PSS, with special focuses on the site of shunt blockade and the postoperative evolution of PSS. The occlusion site was divided according to the location of the blockade with respect to the shunt flow, that is, the inflow, shunt* per se*, and the outflow. The management results were classified according to whether PSS disappeared or not after the shunt occlusion. The former was described as collapsed and the latter as persistent.

### 2.3. Understanding the Pathophysiology of PSS

The possible explanations regarding the pathogenesis of PSS were deduced by applying the circuit theory to the reported management results, including our own case reported elsewhere [7].

### 2.4. Suggestions to PSS Classification

We suggested that PSS be classified according to the two distinct underlying mechanisms, the increase in portal venous pressure (*V*_PV_) and the decrease in shunt resistance (*R*_*S*_).

## 3. Results

### 3.1. Clinical Application of Electric Circuit Model

In normal condition, *R*_*S*_ is sufficiently high and the shunt flow (*I*_*S*_) is negligible, and the basic configuration of PSS model is essentially two resistors connected in series. It is a voltage (=pressure) divider with *R*_*M*_ and portal venous resistance (*R*_*L*_), and the portal pressure (*V*_PV_) can be calculated by the formula *V*_PV_ = *V*_AO_ × {*R*_*L*_/(*R*_*M*_ + *R*_*L*_)}. In other words, portal pressure is directly proportional to portal venous resistance. When a disease process increases the portal venous resistance, such as in liver cirrhosis, portal pressure will increase as well. Therefore the pressure difference across the shunt increases. By Ohm's law, the shunt flow is defined as *I*_*S*_ = *V*_PV_/*R*_*S*_. If *V*_PV_ becomes sufficiently high, *I*_*S*_ can become greater than zero, resulting in PSS formation. The other way for *I*_*S*_ to increase is for *R*_*S*_ to decrease at a fixed *V*_PV_. A clinical example is aneurysmal dilatation of the collateral channel, whether intrahepatic or extrahepatic. Once *R*_*S*_ has decreased, *V*_PV_ also decreases because *R*_*L*_ and *R*_*S*_ are connected in parallel. The portal venous flow (*I*_*P*_) decreases consequently, implicating the portal flow to bypass the liver.

### 3.2. Literature Review

The reported management results of a PSS are presented in Table 1. Most articles described the result as the improvement in the encephalopathic symptoms, not as the morphologic change of the PSS. In 10 cases out of 49 reviewed (20.4%), the morphologic evolution of the PSS was identified. PSS had disappeared or collapsed in 7 cases, whilst in 3 cases, PSS persisted or thrombosed after the occlusion of the shunt by various modalities. Of note, there was no case in which PSS persisted after inflow occlusion, while there were two reported cases in which PSS had collapsed after outflow occlusion.

### 3.3. Understanding the Pathophysiology of a PSS

The cause of a PSS can be deduced by combining the shunt blockade site and the treatment results (Table 2). When a PSS was formed by the increase in *V*_PV_, the evolution of PSS after treatment would vary according to the occlusion site. If the inflow (*ⓐ* in Figures 1 and 2) is blocked, PSS will collapse because the pressure difference across PSS is zero. On the other hand, if the outflow (*ⓒ* in Figures 1 and 2) is blocked, PSS will persist because the pressure across the PSS is *V*_PV_. When a shunt occlusion is made within the shunt channel (*ⓑ* in Figures 1 and 2), the PSS portion proximal to the blockade will persist, whilst that distal to the blockade will collapse. However, when a shunt was formed by the decrease in *R*_*S*_, the PSS would collapse after the shunt blockade. This is irrespective of the occlusion site because *R*_*S*_ becomes infinite.

### 3.4. Suggestions to PSS Classification

PSS can be classified by its underlying causes. The PSS formed by the increase in *V*_PV_ can be classified as portal hypertensive, and the PSS formed by the decrease in *R*_*S*_ can be classified as spontaneous; the shunt channel was opened without the increase in *V*_PV_.

## 4. Discussion

PSS is defined as a condition whereby the gut venous system flows directly to a systemic vein, thus bypassing the liver [16]. The inflow can originate from portal venous systems including the intrahepatic portion of the left portal vein [2, 3]. The draining vein can be a hepatic vein, ductus venosus, an umbilical or paraumbilical vein, or other systemic veins [2, 17]. A shunt implies flow and can be simulated using an electric circuit just like other flow systems [18, 19]. The shunt flow is determined by the formula *I*_*S*_ = *V*_PV_/*R*_*S*_, where *V*_PV_ is portal pressure or the portosystemic pressure gradient, assuming that the systemic venous pressure is ~0 mmHg, and *R*_*S*_ is shunt resistance, which is inversely proportional to the area of the shunt vessel [6]. For a PSS to form, either *V*_PV_ has to increase or *R*_*S*_ has to decrease, or both. When a PSS is formed by an increase in *V*_PV_ as a consequence of increased hepatic resistance *R*_*L*_, *V*_PV_ will continue to increase until collateral vessels dilate or new shunt channel appears [2]. Representative clinical conditions in which *R*_*L*_ is increased are liver cirrhosis and Budd-Chiari syndrome [6, 20]. An extreme case would be congenital absence of portal vein, where *R*_*L*_ = *∞*, *I*_*P*_ = 0, and *I*_*M*_ = *I*_*S*_ [21]. *R*_*L*_ and *R*_*S*_ are inversely related at fixed *I*_*M*_( = *I*_*P*_ + *I*_*S*_), meaning that an increase in *R*_*S*_ by occluding the PSS will result in the increase in *V*_PV_, which in turn increases *I*_*P*_, portal flow through the liver [22]. This can be understood by the same mechanism as the formation of a PSS, but in the reverse direction. Alternately, for *R*_*S*_ to decrease, either shunt vascular diameter must be increased or multiple shunt channels must be opened [23]. *R*_*S*_ can decrease until *I*_*S*_ = *I*_*M*_, with resultant total steal of portal flow though the shunt (*I*_*P*_ = 0). Congenital PSS with or without an aneurysm is a representative clinical condition [24, 25]. Whatever the initiating event may be, either the increase in *V*_PV_ or decrease in *R*_*S*_, once the shunt flow is established the shunt channel can be dilated and even form an aneurysm according to Laplace's law [26].

The electric circuit PSS model can be used to interpret other clinical conditions. For example, we had assumed that the mesenteric vascular resistance *R*_*M*_ was constant. However, there are diseases in which *R*_*M*_ is decreased, such as mesenteric arteriovenous malformation or fistula. Being a pressure divider with *R*_*M*_ and *R*_*L*_, the decrease in *R*_*M*_ has the same effect as the increase in *R*_*L*_, and portal hypertension ensues [27, 28].

Unfortunately, the evolution of a PSS after blockade was not always available in the literature. Two cases have been issued on intrahepatic PSS managed by outflow occlusion, both of which reported the disappearance of PSS [7, 13]. The patients had no liver cirrhosis. On the other hand, one patient who had extrahepatic PSS and liver cirrhosis was managed by outflow occlusion; PSS persisted [14]. Another patient without portal hypertension had patent ductus venosus, and the shunt thrombosed but did not collapse after shunt blockade, probably because the anomaly persisted even when the shunt was blocked [15]. These findings support the notion that intrahepatic PSS occurs in patients without portal hypertension and that it can be congenital or spontaneous in origin, whereas extrahepatic PSS develops as a consequence of portal hypertension [2, 29]. Even in patients who have portal hypertension and intrahepatic PSS together, one condition may provoke the other, because the probability of them to occur simultaneously is low [30]. Also, the reported cases comply with our inference that the cause of a PSS can be deduced after outflow occlusion. At present, both proposed scenarios pertaining to the cause of PSS formation, namely pressure-first (increase in *V*_PV_) and shunt-first (decrease in *R*_*S*_), seem plausible, and published evidence supports both scenarios [2, 3].

Many authors have tried to define types of PSS with different schemes [3, 5, 31]. One of the most confusing terms is “spontaneous,” because it is controversial whether a portal hypertensive PSS should be included in spontaneous PSS or not [30, 32]. It is clear from the electric circuit PSS model that there are two mechanisms underlying a PSS formation, and we suggest the PSS should be classified as portal hypertensive (increase in *V*_PV_) and spontaneous (decrease in *R*_*S*_), to emphasize that the spontaneous PSS patients are without portal hypertension. Finally, the PSS model has clinical implications that when blocking a portal hypertensive PSS, the outflow should not be occluded, because the portal pressure can further increase which may result in severe portal hypertension and bowel congestion [4].

## 5. Conclusions

By simulating PSS using an electric circuit, we found that similarities between the two “flow” systems provide valuable insight to the mechanisms underlying PSS formation. The simulation is simple, easy to understand, and readily applicable to various clinical situations which are seemingly complicated. The shunt blockade site should be selected according to the cause of the PSS because serious complications can occur. Further clinical experiences are required to refine the PSS classification scheme.

## Figures and Tables

**Table 1:** Reported case summary of portosystemic shunt according to shunt blockade type and location.

Authors	Liver cirrhosis	Shunt location	Block site (modality)	Result
Hiraoka et al. [8]	No	Intrahepatic	Inflow (embolization)	Collapsed
Lee et al. [9]	No	Intrahepatic	Inflow (embolization)	Collapsed
Chagnon et al. [10]	No	Intrahepatic	Shunt *per se* (resection)	Collapsed
Lee et al. [9]	No	Intrahepatic	Shunt *per se* (embolization)	Collapsed
Shimoda et al. [11]	Yes	Extrahepatic	Shunt *per se* (surgical closure)	Collapsed
Cauchy et al. [12]	Yes	Extrahepatic	Shunt *per se* (surgical closure)	Persistent
Machida et al. [13]	No	Intrahepatic	Outflow (graft insertion)	Collapsed
Kwon et al. [7]	No	Intrahepatic	Outflow (surgical closure)	Collapsed
Seman et al. [14]	Yes	Extrahepatic	Outflow (surgical closure)	Persistent
Hara et al. [15]	No	Intrahepatic (patent ductus venosus)	Outflow (surgical closure)	Persistent

**Table 2:** The relationship between the location of shunt blockade and the expected fate of portosystemic shunt according to the cause of shunt formation.

Cause	Location of blockade	
	Inflow	Outflow
Increase in portal pressure	Collapse	Persistent
Decrease in shunt resistance	Collapse	Collapse
